# Molecular and biochemical correlates of frontal lobe white matter degeneration in humans with alcohol use disorder

**DOI:** 10.3389/adar.2026.15431

**Published:** 2026-02-24

**Authors:** Suzanne M. de la Monte, Ming Tong, Greg Sutherland

**Affiliations:** 1 Departments of Pathology and Laboratory Medicine, Neurology, Neurosurgery, and Medicine, Rhode Island Hospital, Brown University Health, Alpert Medical School of Brown University, Providence, RI, United States; 2 Department of Medicine, Rhode Island Hospital, Brown University Health, Alpert Medical School of Brown University, Providence, RI, United States; 3 Charles Perkins Centre and School of Medical Sciences, Faculty of Medicine and Health, The University of Sydney, Camperdown, NSW, Australia

**Keywords:** alcohol, alcohol use disorder, gliosis, human, insulin/IGF, white matter, oligodendrocyte

## Abstract

**Background:**

Alcohol-related brain damage caused by heavy alcohol misuse is associated with cognitive-motor impairment and white matter (WM) degeneration. Oligodendrocytes and myelin are major targets, but the underlying mechanisms remain incompletely characterized, particularly in humans.

**Purpose:**

This study investigates the nature of oligodendrocyte dysfunction in anterior frontal lobe tissue from humans with alcohol use disorder (AUD), focusing on molecular and biochemical pathologies that may underlie WM ARBD.

**Methods:**

Cores of fresh frozen human postmortem frontal lobe WM from adults with AUD or no history of substance use disorder (N = 6/group) were analyzed with duplex enzyme-linked immunosorbent assays, multiplex immunoassays, and multiplex RNA hybridization panels.

**Results:**

AUD anterior frontal lobe WM tissue exhibited myelin loss with significant changes in oligodendrocyte/myelin glycoprotein immunoreactivity and mRNA expression, increased glial fibrillary acidic protein, and reduced expression of mRNA transcripts encoding upstream components of the insulin and insulin-like growth factor networks, aspartyl-asparaginyl-β-hydroxylase, and the Notch signaling pathway. In contrast, neuroinflammatory mediators and Alzheimer’s disease (AD) biomarkers were largely unaffected.

**Conclusion:**

Human AUD anterior frontal lobe WM pathology is accompanied by significant alterations in oligodendrocyte and astrocyte function, with alterations in Notch and insulin/IGF signaling. The findings provide new information on the mechanisms of AUD-mediated WM degeneration as well as potential strategies for diagnosing ARBD in humans.

## Introduction

Alcohol use disorder (AUD) is caused by chronic heavy or repeated binge alcohol misuse, resulting in cognitive-motor and executive dysfunctions [1]. AUD leads to brain atrophy [2] with notable targeting of white matter (WM) [3], particularly in the corpus callosum [4, 5], frontal lobe, temporal lobe, and cerebellum [6]. Ethanol’s neurotoxic and degenerative effects [6] on gray matter/cortical structures compromise neuronal survival, neurotransmitter functions, and synaptic plasticity, whereas in WM, the main targets are oligodendrocytes and myelinated fibers [7, 8]. Together, these complex pathophysiologic processes comprise the substrate of alcohol-related brain damage (ARBD). AUD’s adverse effects on frontal lobe functions are manifested by impairments in abstract thinking and planning, visuospatial functions, and organizational capacity. However, the mechanistic and molecular/biochemical drivers of WM pathology that mediated neurobehavioral dysfunctions and can progress to dementia in humans are poorly understood.

White matter (WM), which occupies nearly 50% of the brain’s volume, is particularly vulnerable to acute, subacute, and chronic alcohol-mediated injuries [6] due to the targeting of oligodendrocytes, myelin, supporting cells, and axons [7, 8]. Experimentally, alcohol-mediated injury to oligodendrocytes impairs metabolic, homeostatic, survival, and myelin synthesis/maintenance functions [9]. Attendant loss of mature myelin and eventually axons compromises neuronal conductivity and synaptic integrity [5, 10], contributing to cognitive impairment [6, 11]. The severity of WM atrophy in ARBD correlates with maximum daily and lifetime alcohol exposure [6, 12, 13], indicating that alcohol dose governs clinical outcomes. However, other factors can modulate disease progression and permanence since chronic ARBD tends to be only partially reversed by abstinence [14–17], suggesting that active interventions are required to curtail or reverse chronic ARBD. The knowledge gap in understanding WM ARBD is due to inadequate research, as shown by the disproportionately low number of NCBI/PubMed articles published between 1960 and 2025 related to alcohol’s effects on WM (2134) or the cellular, biochemical, and molecular pathologic effects on oligodendrocytes (729) and myelin (477) compared with neurons (23560). Similarly, alcohol-related brain research publications on astrocyte (1872) and microglial (978) pathologies also lag. Improved understanding of ARBD’s cellular pathologies could lead to novel therapeutics to reduce cognitive impairment and disability.

Clues to understanding the pathogenesis of WM degeneration in ARBD stemmed from studies showing that chronic ethanol exposures alter myelin lipid composition, resulting in prominent reductions in sulfatides and sphingomyelins [17, 18]. Those findings provided definitive evidence that oligodendrocyte functions are impaired in ARBD. Besides lipids, oligodendrocytes synthesize maturation- and function-related structural integral membrane proteins, including myelin basic protein (MBP), myelin associated glycoprotein (MAG), myelin oligodendrocyte glycoprotein (MOG), proteolipid protein (PLP) [19]. Correspondingly, preclinical studies have shown that oligodendrocyte/myelin glycoprotein expression is also adversely impacted by alcohol exposure [20, 21]. Mechanistically, experimental ARBD’s inhibitory effects on WM integrity and oligodendrocyte function have been linked to impaired signaling through insulin/insulin-like growth factor, type 1 (IGF-1)-Akt pathways [22, 23] and downstream via the mechanistic target of rapamycin (mTOR) [24, 25], which is critical to oligodendrocyte differentiation and stage-specific antigen and myelin protein expression [26]. Correspondingly, the loss of mTOR/mTORC2 signaling in oligoprogenitor cells reduces myelination [27], myelin thickness, axonal density, and oligodendrocyte populations, and increases gliosis [28].

Notch networks represent another critically important signaling pathway for regulating oligodendrocyte function [29, 30]. During development, ethanol’s inhibitory effects on Notch were shown to be associated with significantly reduced expression of aspartyl-asparaginyl-β-hydroxylase (ASPH) [31, 32], which is regulated by insulin/IGF-Akt [32, 33]. ASPH has known roles in the activation of Notch pathways; its effects are mediated by the hydroxylation of specific EGF-like domains within Notch. Subsequent cleavage of the protein releases the Notch intracellular domain, which translocates to the nucleus and functions as a transcription factor for hairy and Enhancer of split homologs (HES) and Hairy/enhancer-of-split related with YRPW motif protein (HEY) [31, 34]. Recent experimental models have demonstrated that ASPH is expressed in WM and that chronic ethanol exposure inhibits both ASPH and Notch [35]. Therefore, the inhibitory effects of ethanol on insulin/IGF-Akt signaling impair ASPH’s crosstalk with Notch networks. It is noteworthy that the potential roles of impaired insulin/IGF signaling, ASPH expression, and Notch activation as mediators of WM atrophy and oligodendrocyte loss stemmed from human studies of cerebral autosomal dominant arteriopathy with subcortical infarcts and leukoencephalopathy (CADASIL) [36].

The present study was designed to assess the extent to which WM ARBD in humans exhibits molecular and biochemical abnormalities like those observed in experimental models, including biomarkers of oligodendrocyte/myelin dysfunction, impaired insulin/IGF signaling, and inhibition of ASPH-Notch signaling. In addition, given the current interest in determining the potential role of Alzheimer’s disease (AD) as a mediator of cognitive impairment in ARBD, the studies included measurements of AD biomarkers in brain tissue. We recently published a related study of AUD’s effects on cerebellar vermis white matter using the same human subject cohort [37]. The dual effort was launched because the anterior frontal lobe and cerebellar vermis are distinct characteristic targets of brain atrophy in humans with AUD [38], suggesting that their responses to chronic alcohol exposure might also differ.

## Materials and methods

Materials and instruments

The sources of materials, reagents, and instruments used in this research are listed in (Supplementary Table S1).

### Source of human brain tissue samples

Fresh frozen human postmortem tissue blocks from the superior anterior frontal lobe (Brodmann Area 8/9), including cortex and underlying white matter from volunteer donors with or without a clinical diagnosis of alcohol use disorder (AUD) (n = 6/group), were obtained from the New South Wales Brain Tissue Research Centre (BTRC) in Sydney, Australia [12, 39]. The BTRC Scientific Advisory Committee, University of Sydney Human Research Ethics Committee (2018/HE000477), and Brown University Health Institutional Review Board (CMTT/PROJ:#013024) approved this human tissue research. The cases included in this study are the same as those used to characterize chronic AUD effects on the cerebellar vermis [37]. Apart from alcohol, the donors had no other substance use disorder, and they had not participated in clinical trials. The salient demographic, clinical, and postmortem statistics are provided in Table 1. The most notable differences were the greater lifetime quantity of alcohol consumed (p < 0.0001), and lower mean brain weight (p = 0.02) in the AUD group.

**TABLE 1:** Human subjects-postmortem brain tissue donors.

Characteristics	AUD	Controls	P-value
# Cases	6	6	​
Age (Years)	57.33 ± 7.37	57.83 ± 6.68	N.S.
Age (range-years)	50–70	50–69	N.S.
Male/Female (#)*	6M/0F	6M/0F	N.S.
Drinking history (Years)	34.0 ± 7.11	26.2 ± 4.27	0.043
Lifetime alcohol (kg)	2,784 ± 1,465	42.3 ± 35.5	0.003
RIN	6.84 ± 1.35	7.50 ± 0.70	N.S.
Phosphatidylethanol (PEth)	2.19 ± 3.08	20.4 ± 29.44	N.S.
Smoking history (Y/N)*	4/6	3/6	N.S.
Postmortem interval (hours)	32.17 ± 24.81	22.17 ± 6.43	N.S.
Brain pH	6.66 ± 0.23	6.61 ± 0.21	N.S.
Brain weight (g)	1,335 ± 133	1,505 ± 115	0.02
Liver disease (Y/N)*	4/6	1/6	*0.079*

Characteristics of alcohol use disorder (AUD) and control deceased donors. Data corresponds to either counts (#) or mean ± S.D., Intergroup comparisons of mean values were made using Welsh t-tests. *Proportional differences were compared using Chi-square tests. N.S., not statistically significant. Italicized p-value represents a statistical trend (0.05 < p < 0.10).

### Tissue processing and homogenization

To demonstrate the effects of AUD on WM myelin integrity, frontal lobe tissue fixed in 10% neutral buffered formalin and embedded in paraffin was examined in histological sections stained with Luxol fast blue, hematoxylin, and eosin. For molecular and biochemical assays, duplicate 100 mg samples of fresh frozen frontal lobe were homogenized using a TissueLyser II instrument (Qiagen, Germantown, MD, USA) with 5-mm diameter stainless steel beads to achieve thorough tissue disruption. To generate samples for protein analysis, the tissues were homogenized in buffer contained 50 mM Tris (pH 7.5), 150 mM NaCl, 5 mM EDTA (pH 8.0), 50 mM NaF, 0.1% Triton X-100), protease inhibitor cocktail (1mM PMSF, 0.1 mM TPCK, 2 μg/mL aprotinin, 2 μg/mL pepstatin A, 1 μg/mL leupeptin, 1 mM NaF, 1 mM Na_4_P_2_O_7_), and 10 mM Na_3_VO_4_ to inhibit phosphatases. Cellular debris was pelleted by centrifugation (14,000 rpm × 10 min at 4 °C), and the clarified supernatants were stored at −80 °C. The bicinchoninic acid (BCA) assay was used to measure protein concentration. To isolate total RNA, duplicate fresh frozen tissue samples (50 mg each) were homogenized in QIAzol Lysis Reagent (Qiagen, Germantown, MD, USA) using the TissueLyzer II with 5-mm stainless steel beads, following the manufacturer’s protocol^1^.

### Duplex enzyme-linked immunosorbent assay (ELISA)

A duplex ELISA format was used to measure immunoreactivity and to enable comparisons across molecules and subject groups by normalizing the expression levels of specific molecules to an internal control [35, 40]. For these studies, immunoreactivity was normalized to large acidic ribosomal protein (RPLPO) [41], which increases linearly with protein concentration and is generally not modulated by disease state [42]. The molecules assayed included: immature and pre-myelinating oligodendrocyte glycoproteins (2′,3′-cyclic nucleotide 3′phosphodiesterase (CNPase), proteolipid protein 1 (PLP), Platelet-derived growth factor receptor, alpha peptide (PDGFRA), Group-specific component Vitamin D Binding (GALC)); mature myelinating oligodendrocyte glycoproteins (myelin-associated glycoprotein 1 (MAG), myelin oligodendrocyte glycoprotein (MOG), myelin basic protein (MBP)); astrocyte markers (nestin, vimentin, and glial fibrillary acidic protein (GFAP)); and AD and related biomarkers (Amyloid beta precursor protein (AβPP), Amyloid beta (Aβ_1-42_), Tau, pTau (PHF13; S396), ubiquitin, and 4-hydroxynonenal (HNE)). See Supplementary Table S2 for the antibody sources, concentrations used, and research resource identifier (RRID) numbers, and Supplementary Table S3 for the functions the glial markers assessed by duplex ELISA or multiplex RNA hybridization (see below).

The procedures used for duplex ELISAs were previously described [35, 40]. In brief, triplicate 50 ng protein samples in bicarbonate binding buffer were robotically pipetted (Eppendorf EpMotion 330, Framingham, MA, USA) into 96-well MaxiSorp plates. Following overnight adsorption, masking of non-specific binding sites with Superblock TBS, and incubation with primary antibodies (0.2–5.0 μg/mL), immunoreactivity detected with horseradish peroxidase (HRP)-conjugated secondary antibodies and Amplex UltraRed soluble fluorophore, was measured (Ex 530 nm/Em 590 nm) in a Spectra-Max M5 Multimode Plate Reader (Molecular Devices, Sunnyvale, CA, USA). Subsequent measurement of RPLPO immunoreactivity was achieved by incubating Tris-buffered saline (TBS)-rinsed wells with biotin-conjugated anti-RPLPO, followed by streptavidin-conjugated alkaline phosphatase, and 4-Methylumbelliferyl phosphate (4-MUP) (Ex 360 nm/Em 450 nm). Fluorescence was measured in a SpectraMax M5. Inter-group statistical comparisons were made with the calculated ratios of target protein to RPLPO immunoreactivity.

### Multiplex ELISAs

Commercial magnetic bead-based 11-Plex plus 10-Plex MILLIPLEX MAP panels were used to measure human cytokines/chemokines (Millipore, Burlington, MA, USA)^2^ (Supplementary Table S4), and Akt and phospho-Akt pathway 7-Plex Multispecies Panels (LHO0001M Lot 1012019) (ThermoFisher/Invitrogen, Camarillo, CA USA) (Supplementary Table S5). In accordance with the manufacturers’ multiplex ELISA protocols, antigens partitioned in the clarified protein homogenates were captured by incubation with antibody-coated beads. Fluorescent immunoreactivity was measured in a Luminex MAGPIX instrument (Diasorin, Austin TX USA) with xPONENT software following sequential incubations with biotinylated secondary antibodies and phycoerythrin-conjugated streptavidin. Immunoreactivity was calculated against standard curves generated with MAGPIX calibration and verification standards for each analyte.

### Quantigene 2.0 RNA multiplex assay

Gene expression corresponding to human glial mRNA transcripts was measured with a custom 10-Plex Human Glial panel (QuantiGene 2.0 Plex Set Cat# 312185) (Supplementary Table S6). Insulin/Notch pathway mRNA expression was measured with a custom 20-plex assay (Affymetrix Inc., Santa Clara, CA, USA, Cat# 312177) (Supplementary Table S7). The RNA hybridization format enabled direct mRNA quantification using xMAP Luminex beads with reporter signal amplification and simultaneous measurement of RPL13a as the internal normalizing control^3^. The 96-well plate assays were performed by first incubating 1-µg samples of total RNA with xMAP fluorescent capture beads together with blocking reagent and RNA oligonucleotide pre-amplifier, amplifier, and biotin-labeled probe sets. Next, the samples were incubated with streptavidin-conjugated R-Phycoerythrin. Fluorescent signals were subsequently detected with a Luminex MAGPIX instrument (Diasorin, Austin, TX USA). After subtracting probe-related background from the target median fluorescence intensity, the results were normalized to RPL13a. MAGPIX calibration and verification standards were used throughout to ensure the Streptavidin-Phycoerythrin fluorescence levels were proportional to RNA transcript abundance captured by the beads.

### Data analysis

*The results of this research were generated with frontal lobe samples from 6 AUD and 6 control cases.* The protein and mRNA studies were each conducted using two adjacent tissue cores per case, and all assays were performed in triplicate, under code. Results generated from the triplicate data points were averaged to produce a single value for statistical analysis. GraphPad Prism 10.4 software (GraphPad Software Inc., Boston, MA) was used to generate violin plots displaying the distributions of protein and mRNA expression levels, including the median, first and third quartiles, and upper and lower data points. Multiplex panel data were analyzed using a two-way Mixed-Model Analysis of Variance (ANOVA) with *post hoc* Šídák’s multiple comparison tests. Data for the individual assays passed the D’Agostino & Pearson test for normality (alpha = 0.05). Additionally, between-group comparisons were performed using heatmaps to assess the effects of AUD within the glial, inflammatory, trophic factor, Akt pathway signaling protein biomarker panels, and glial, Insulin pathway, and Notch pathway mRNA biomarker panels. Software-generated statistically significant (p ≤ 0.05) and trend-wise (0.05 < p < 0.10) between-group differences are displayed in the Tables and Graphs. The trendwise p-values are noted only as potentially bordering on significance. However, they are nonsignificant [43] and therefore must be regarded with caution.

## Results

### White matter pathology

**FIGURE 1:**
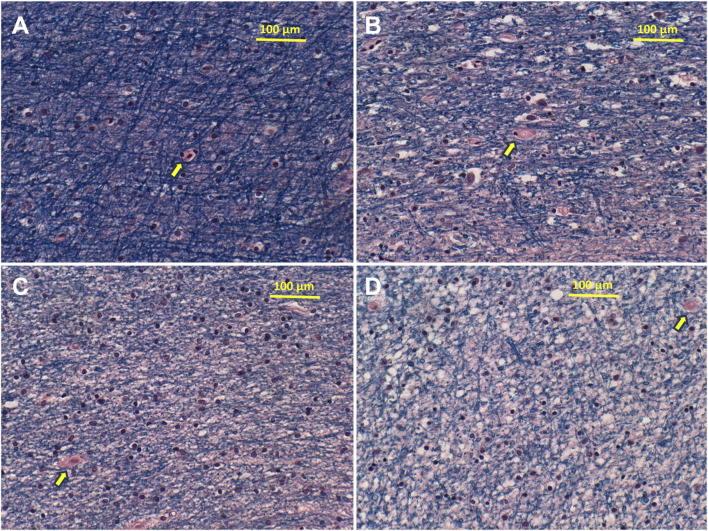
AUD reduces cerebral white matter myelin integrity. Formalin-fixed, paraffin-embedded sections of anterior frontal lobe white matter from **(A)** control and **(B–D)** AUD cases were stained with Luxol fast blue, hematoxylin and eosin. Blue fibrillar staining corresponds to myelin. Reduced blue fibrillar staining reflects loss of myelin. Small round dark nuclei correspond to oligodendrocytes. Yellow arrows show small vessels, which have thickened fibrillar walls in Panels **(B–D)** compared with A. Original magnifications = ×200 (note scale bars).

Histological sections of frontal lobe stained with Luxol Fast Blue, Hematoxylin and Eosin revealed intense and uniform Luxol Fast Blue staining of control myelin and variable degrees of pallor with vacuolation of myelin in AUD cases (Figure 1). In addition, in the AUD cases, small white matter vessels exhibited thickening and fibrosis of the walls with scarcely detectable lumens. Corresponding myelin lipid composition abnormalities consisted of proportionally greater reductions in sphingolipid compared with phospholipid expression (Supplementary Figure S1) as demonstrated previously by imaging mass spectrometry [18]. Since myelin is generated and maintained by oligodendrocytes, it is important to understand how AUD impacts oligodendrocyte function using molecular and biochemical approaches.

### Oligodendrocyte/glial, and neurofilament light chain protein expression

**FIGURE 2:**
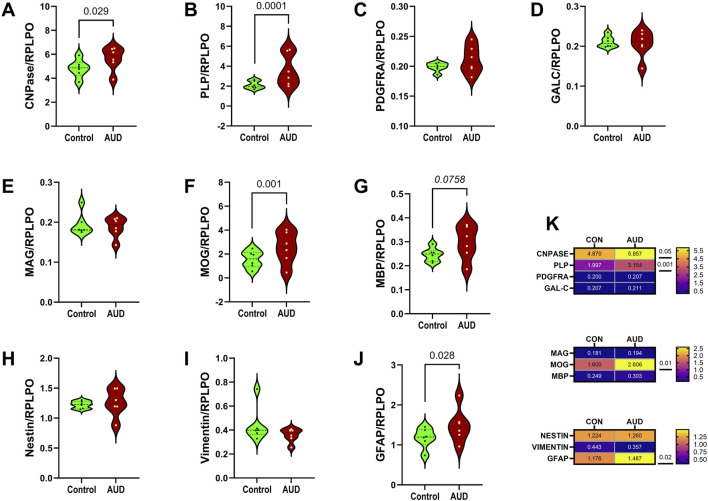
AUD impacts glial protein immunoreactivity in frontal lobe white matter. Postmortem frontal lobe white matter tissue from 6 AUD and 6 control donors was used to extract proteins for immunoassays. Duplex ELISAs measured immunoreactivity to **(A)** CNPase, **(B)** PLP, **(C)** PDGFRA, **(D)** GALC, **(E)** MAG, **(F)** MOG, **(G)** MBP, **(H)** Nestin, **(I)** Vimentin, and **(J)** GFAP with results normalized to RPLPO. Violin plots (mean, quartiles, individual points) display inter-group differences in the levels of immunoreactivity. Data were analyzed by two-way ANOVA (Table 2) and *post hoc* multiple comparisons tests. Significant (p ≤ 0.05) and statistical trend-wise differences (italicized; 0.10 < p < 0.05) are shown within the panels. **(K)** Heatmaps display differences in relative abundance and effects of AUD on immature oligodendrocyte (CNPase, PLP, PDGFRA, and GALC), mature oligodendrocyte (MAG, MOG, MBP), and astrocyte (Nestin, vimentin, GFAP) markers, including results of *post hoc* multiple comparisons tests.

Duplex ELISAs measured immature (CNPase, PLP, PDGFRA, GALC) and mature (MAG, MOG, MBP) oligodendrocyte proteins, and astrocyte proteins (Nestin, vimentin, GFAP) (Supplementary Table S3). The classification of oligodendrocyte-myelin glycoproteins was established based on the timing and stages of oligodendrocyte differentiation, as well as developmental or post-injury differentiation [30, 44–48]. Immunoreactivity was normalized to RPLPO. Two-way ANOVA detected significant AUD and AUD x biomarker interactive effects on immature myelin/oligodendrocyte protein expression, and significant biomarker effects on immature oligodendrocyte, mature oligodendrocyte, and astrocyte biomarkers (Table 2). Post-hoc multiple comparison tests revealed significantly higher levels of CNPase (Figure 2A), PLP (Figure 2B), MOG (Figure 2F), and GFAP (Figure 2J), and a statistical trendwise increase in MBP (Figure 2G) in AUD versus control frontal lobe WM. In contrast, there were no significant or trendwise effects of AUD on PDGFRα (Figure 2C), GALC (Figure 2D), MAG (Figure 2E), Nestin (Figure 2H), or vimentin (Figure 2I). Heatmaps comparing the differences in protein expression showed that the dominant effects of AUD mainly occurred with respect to the more abundant oligodendrocyte/myelin glycoproteins, whereas for low-abundance molecules, the impact of AUD was nil (Figure 2K).

**TABLE 2:** Frontal lobe-two-way ANOVA duplex and multiplex ELISA results.

Biomarker	AUD-factor F-ratio	p-value	Biomarker F-ratio	p-value	AUD x biomarker F-ratio	p-value
Oligodendrocyte/myelin biomarkers						
Immature myelin	**7.673**	**0.0085**	**131.1**	**<0.0001**	**2.956**	**0.0438**
Mature myelin	2.676	N.S.	**33.51**	**<0.0001**	2.351	N.S.
Astrocyte	1.256	N.S.	**57.73**	**<0.0001**	2.264	N.S.
Cytokines/chemokines						
Proinflammatory cytokines	0.012	N.S.	**24.45**	**<0.0001**	0.05	N.S.
Proinflammatory chemokines	0.0254	N.S.	**20.70**	**<0.0001**	0.540	N.S.
Anti-inflammatory	**6.453**	**0.0147**	**1,184**	**<0.0001**	0.480	N.S.
Trophic factors and AD neurodegeneration						
Trophic factors	**4.463**	**0.0365**	**1715**	**<0.0001**	**4.701**	**0.0006**
​	​	​	​	​	​	​
AKT pathway						
Total protein	0.001	N.S.	**72.78**	**<0.0001**	0.011	N.S.
Phosphoproteins	0.059	N.S.	**17.20**	**<0.0001**	0.056	N.S.
P/T relative phosphorylation	2.167	N.S.	**48.70**	**<0.0001**	1.868	N.S.
AD neurodegeneration						
AD biomarkers	0.005	N.S.	**108.5**	**<0.0001**	0.1708	N.S.

Anterior frontal lobe tissue samples from human alcohol use disorder (AUD) or control deceased participants (n = 6/group) were analyzed by duplex or multiplex ELISAs (see Methods and Supplementary Tables S2, S4, S5 for biomarker descriptions). The results were analyzed by two-way ANOVA, comparing the effects of AUD, clustered biomarkers, and AUD x Biomarker interactions. The calculated F-ratios and p-values corresponding to the ANOVA, results are indicated. Significant effects (p ≤ 0.05) are highlighted with bold font. N.S., not significant. The results of *post hoc* multiple comparisons tests are shown in Figures 2, 5, 6, Supplementary Figure S2, S3. Bold font indicates significant differences.

### Oligodendrocyte/glial mRNA expression

**FIGURE 3:**
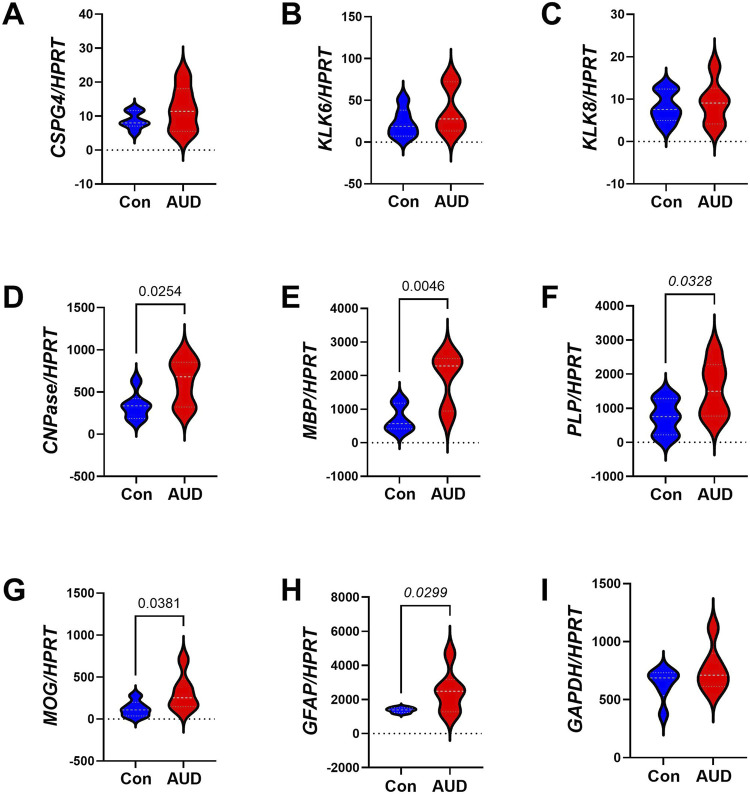
AUD alters glial mRNA expression in frontal lobe white matter. Total RNA isolated from frontal lobe tissue from 6 AUD and 6 control cases was used to measure gene expression using a custom multiplex magnetic bead-based RNA hybridization assay (see Methods). Violin plots (mean, quartiles, individual points) display inter-group differences in the levels of gene expression corresponding to **(A)** *CSPG4*, **(B)** *KLK6*, **(C)** *KLK8*, **(D)** *CNPASE*, **(E)** *MBP*, **(F)** *PLP*, **(G)** *MOG*, **(H)** *GFAP*, **(I)** *GAPDH*, with results normalized to *HPRT*. Data were analyzed by two-way ANOVA (Table 2) and *post hoc* multiple comparisons tests. Significant (p ≤ 0.05) differences are shown within the panels. Also see heatmap in Figure 4A.

**FIGURE 4:**
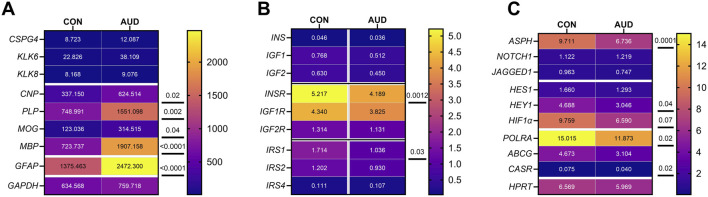
Gene expression heatmaps corresponding to the effects of AUD on **(A)** glial, **(B)** insulin/IGF pathway, and **(C)** Notch pathway mRNAs. Total RNA was isolated from postmortem frontal lobe tissue from 6 AUD and 6 control donors. mRNA expression was measured with custom multiplex magnetic bead-based RNA hybridization assays. The data displayed within the cells reflects mRNA expression normalized to the HPRT control gene (see Methods). Data were analyzed by two-way ANOVA (Table 2) and *post hoc* multiple comparisons tests. Also see Figures 7, 8.

To determine whether the effects of AUD were mediated at the level of transcription, a custom multiplex RNA hybridization assay was used to measure glial gene expression, with *HPRT* as a housekeeping control. The mRNA transcripts corresponded to Chondroitin Sulfate Proteoglycan 4 (*CSPG4),* Kallikrein-related peptidase 6 *(KLK6), KLK8, CNPase*, *PLP, MOG, MBP*, *GFAP,* and glyceraldehyde-3-phosphate dehydrogenase *(GAPDH;* control gene*)*. Two-way ANOVA demonstrated significant AUD (p < 00,001), biomarker (p < 0.0001), and AUD x biomarker interactive (p = 0.0013) effects on glial gene expression Table 3. Post hoc multiple comparison tests revealed significantly higher levels of *CNPase* (Figure 3D), *MBP* (Figure 3E), *PLP* (Figure 3F), *MOG* (Figure 3G), and *GFAP* (Figure 3H) in AUD compared to control samples. In contrast, there were no significant inter-group differences in the mean levels of *CSPG4* (Figure 3A), *KLK6* (Figure 3B), *KLK8* (Figure 3C), or *GAPDH* (Figure 3I). The corresponding heatmap depicts clustered upregulated expression of predominantly mature oligodendrocyte/myelin genes as well as *GFAP*, corresponding to an astrocytic response (Figure 4A). In contrast, mRNA transcripts encoding immature oligodendrocyte/myelin genes were mainly expressed at low levels in both groups. The modest AUD-associated elevations in *CSPG4, KLK6*, and *KLK8* did not reach statistical significance.

**TABLE 3:** Frontal lobe-two-way ANOVA multiplex mRNA results.

mRNA	AUD-factor F-ratio	p-value	Biomarker F-ratio	p-value	AUD x biomarker F-ratio	p-value
Glial genes	**23.32**	**<0.0001**	**28.55**	**<0.0001**	**3.545**	**0.0013**
Insulin-IGF-IRS	**11.45**	**0.0011**	**120.9**	**<0.0001**	1.187	N.S.
Notch pathway	**16.85**	**<0.0001**	**66.18**	**<0.0001**	1.608	N.S.

Anterior frontal lobe tissue samples from human alcohol use disorder (AUD) or control deceased donors were assayed with custom Quantigene 2.0 Panels to measure mRNA, expression of Glial, Insulin-IGF-IRS, and Notch Pathway Genes (see Methods and Supplementary Tables S3, S5, S6). The results were analyzed by two-way ANOVA, comparing the effects of AUD, biomarkers, and AUD x Biomarker interactions. Calculated F-ratios and p-values corresponding to the ANOVA, results are indicated. The results of *post hoc* multiple comparison tests are shown in Figures 3, 4, 7, 8. Bold font indicates significant differences.

### Inflammatory factor expression

Multiplex ELISAs were used to measure 15 proinflammatory cytokines, proinflammatory chemokines, and anti-inflammatory cytokines. The objective was to assess the role of persistent inflammation in mediating ARBD. The cytokine results, expressed as fluorescent light units (FLU) per 100 µg of protein isolated from brain tissue, were analyzed using a two-way ANOVA with *post hoc* repeated-measures tests. Significant effects of AUD were observed for anti-inflammatory markers, and significant biomarker effects were detected across all 3 categories of inflammatory mediators (Table 2). In contrast, there were no significant or trendwise AUD × cytokine biomarker interactive effects. The results are depicted with a heatmap (Supplementary Figure S2). Post hoc multiple comparisons tests detected a significant reduction in anti-inflammatory interleukin-10 (IL-10) in the AUD samples. The other 14 cytokines/chemokines were either similarly expressed in AUD and control samples or showed intergroup differences that did not reach statistical significance due to large standard deviations.

### Trophic factors

**FIGURE 5:**
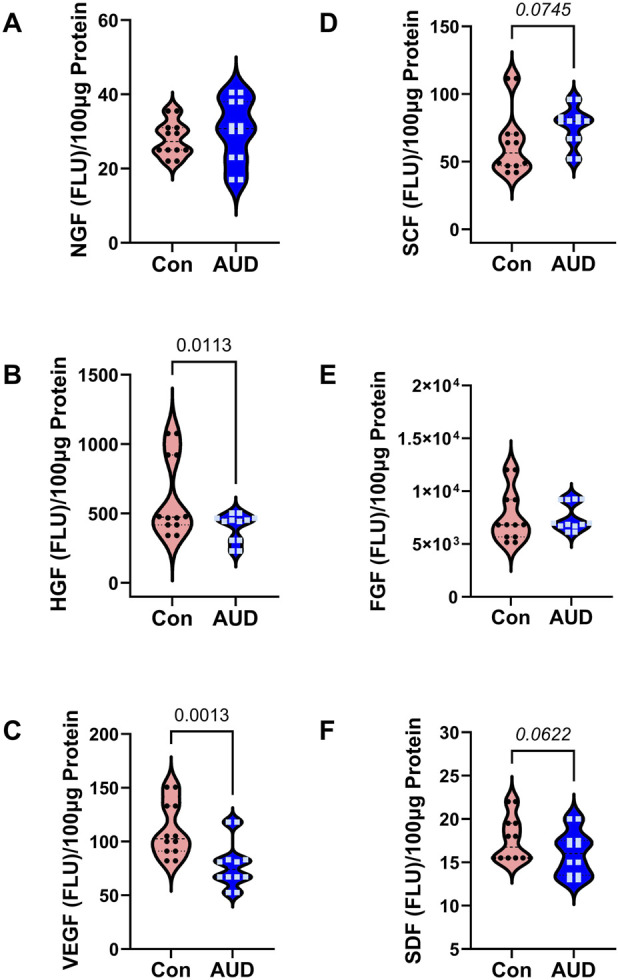
AUD affects trophic factor immunoreactivity in frontal lobe white matter. Immunoreactivity was measured in proteins extracted from postmortem brain tissue of 6 AUD and 6 control donors. Immunoreactivity (FLU = fluorescent light units) to **(A)** NGF, **(B)** HGF, **(C)** VEGF, **(D)** SCF, **(E)** FGF, and **(F)** SDF was measured in 100 µg protein samples using a magnetic bead-based multiplex ELISA. Violin plots (mean, quartiles, individual points) display inter-group differences. Data were analyzed by two-way ANOVA (Table 2) and *post hoc* multiple comparisons tests. Significant (p ≤ 0.05) and statistical trend-wise differences (italicized; 0.10 < p < 0.05) are shown within the panels.

**FIGURE 6:**
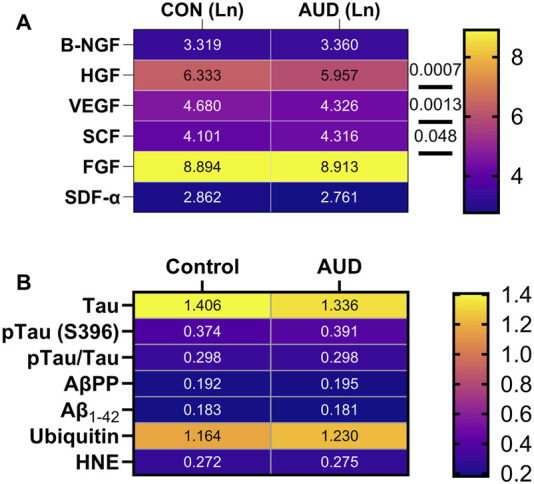
Heatmaps illustrate AUD effects and within-group differences in the relative expression of **(A)** trophic factors and **(B)** AD biomarkers in the anterior frontal lobe. Samples were obtained from 6 AUD and 6 control donors. Trophic factor immunoreactivity was measured using a multiplex ELISA panel (See Figure 5), and the AD biomarkers (Tau, phosphorylated-Tau (S396), amyloid-beta precursor protein (AβPP), Aβ_1-42_, ubiquitin, and 4-hydroxynonanal (HNE)) were measured by Duplex ELISA with results normalized to RPLPO (see Methods). In addition, the calculated relative levels of Tau phosphorylation (pTau/Tau) results are displayed. The data were analyzed using a two-way ANOVA with *post hoc* multiple comparisons. No statistically significant or trendwise differences were detected in the AD biomarkers.

The trophic factors measured by multiplex ELISA included nerve growth factor (NGF), hepatocyte growth factor (HGF), vascular endothelial growth factor (VEGF), stem cell factor (SCF), basic fibroblast growth factor (FGF), and stromal cell-derived factor (SDF). Immunoreactivity was measured in 100 μg samples of frontal lobe protein. Two-way ANOVA demonstrated significant effects of AUD, trophic factor subtype, and AUD x trophic factor interactions (Table 2). Post hoc multiple comparisons detected significantly reduced HGF (Figure 5B) and VEGF (Figure 5C), and significantly increased SCF (Figure 5D) in AUD. In contrast, there were no significant inter-group differences in the levels of B-NGF (Figure 5A), FGF (Figure 5E), or SDF-α (Figure 5F). The inter-group differences were further displayed with a heatmap using Log-transformed data to accommodate the large differences in the trophic factor expression (Figure 6A). It is noteworthy that the combined data shown in Figures 5, 6 suggest that HGF, which has pro-survival and repair functions, VEGF, which has neuroprotective properties, and SDF-1α, which has a vital role in neuro-progenitor cell migratory behavior from the subventricular zone following injury are reduced, and whereas SCF, which promotes neurogenesis angiogenesis and impacts CNS cell differentiation and fate is increased in AUD. In contrast, WM expression of b-FGF and β-NGF was unaffected by AUD, possibly due to their prominent roles in modulating neuronal rather than glial functions.

### Alzheimer’s disease biomarkers

To address the potential role of Alzheimer’s disease (AD) neurodegeneration as a cause or consequence of AUD, the brain samples were used to measure standard biomarkers of AD-type neurodegeneration, including Tau, phospho-Tau (pTau-S396; PHF13), pTau(S396)/Tau, amyloid-beta (Aβ_1-42_), Aβ-precursor protein (AβPP), ubiquitin, and 4-hydroxynonenal (HNE). Immunoreactivity in 50 ng samples of frontal lobe protein was measured by duplex ELISA. The results were normalized to RPLPO. Two-way ANOVA detected significant effects of biomarker factors but no significant effects of AUD, or AUD × biomarker interactions on immunoreactivity (Table 2). The *post hoc* multiple comparisons test failed to detect any significant or trendwise intergroup differences in the levels of AD biomarkers. The heatmap (Figure 6B) shows abundant Tau and ubiquitin immunoreactivity and relatively low levels of pTau (S396), AβPP, Aβ(Aβ_1-42_), and HNE, with no AUD-related effects.

### Insulin/IGF/IRS mRNA expression

**FIGURE 7:**
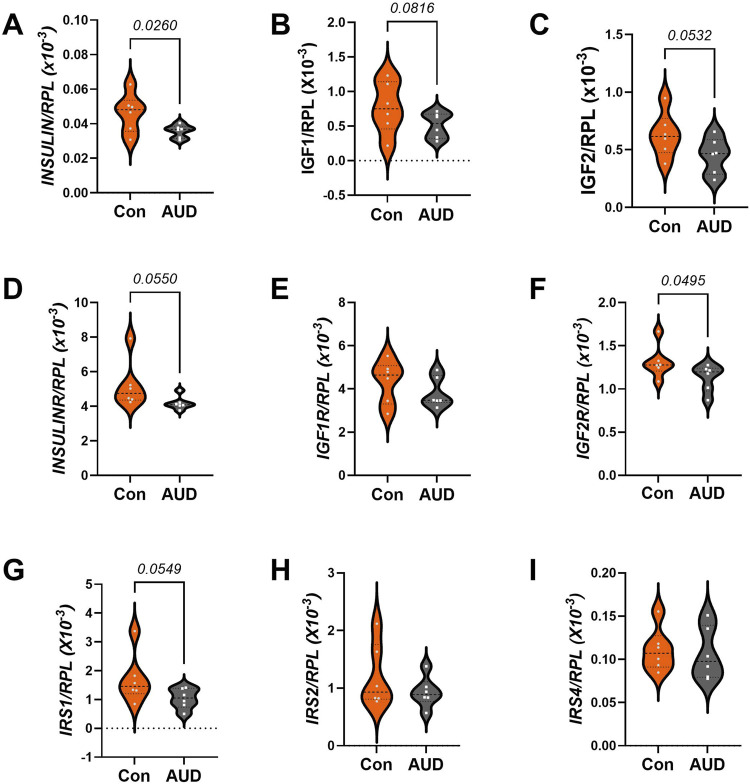
AUD effects on insulin/IGF/IRS pathway mRNA transcripts in frontal lobe white matter. Gene expression was measured in RNA isolated from 6 AUD and 6 control donors using a custom multiplex magnetic bead-based RNA hybridization assay (see Methods). Violin plots (mean, quartiles, individual points) display inter-group differences in the levels of gene expression corresponding to **(A)** *INSULIN*, **(B)** *IGF1*, **(C)** *IGF2*, **(D)** *INSULINR*, **(E)** *IGF1R*, **(F)** *IGF2R*, **(G)** *IRS1*, **(H)** *IRS2*, and **(I)** *IRS4*, with results normalized to *RPL13a*. Data were analyzed by two-way ANOVA (Table 2) and *post hoc* multiple comparisons tests. Significant (p ≤ 0.05) and statistical trend-wise differences (italicized; 0.10 < p < 0.05) are shown within the panels. Also see Figure 4B-Heatmap.

Multiplex RNA hybridization assays compared AUD and control levels of insulin (*INS), insulin-like growth factor 1 (IGF1), IGF2,* insulin receptor *(INSR), IGF1R, IGF2R, insulin receptor substrate, Type 1 (IRS1), IRS2,* and *IRS4* (Supplementary Table S7)*.* The results were normalized to ribosomal protein L13a (*RPL13a*). Intergroup statistical comparisons by two-way ANOVA detected significant effects of AUD and biomarker type, but no AUD x Biomarker interactive effects on gene expression (Table 3). Post hoc multiple comparisons tests demonstrated significantly reduced *INS* (Figure 7A) and *IGF2R* (Figure 7F), and trendwise reductions in *IGF1* (Figure 7B), *IGF2* (Figure 7C), *INSR* (Figure 7D), and *IRS1* (Figure 7G). No inter-group differences were observed for IGF1R (Figure 7E), IRS2 (Figure 7H), or IRS4 (Figure 7I). The broad inhibitory effects of AUD on mRNA transcripts encoding growth factors (*INS, IGF1, IGF2*), growth factor receptors (*INSR, IGF2R*), and IRS1 are shown in both the violin plots (Figure 7) and the corresponding heatmap (Figure 4B).

### Akt pathway analysis

Results obtained with the 7-plex Akt pathway total protein and phosphoprotein panels, and the calculated levels of relative protein phosphorylation (p/T), were analyzed using two-way ANOVA (Table 2). The only significant effects pertained to the biomarker factors (all p < 0.0001); the AUD and AUD x Biomarker interactive effects were not statistically significant. The graphed results corresponding to all the measurements and calculations are shown in Supplementary Figure S3. Post hoc multiple comparison tests detected only one significant inter-group difference related to the relative levels of IGF1-R phosphorylation. Otherwise, there were no intergroup differences corresponding to molecules included in the total Akt (Supplementary Figure S3A) and phospho-Akt (Supplementary Figure S3B) panels, or the calculated p/T ratios (Supplementary Figure S3C).

### Notch pathway mRNA studies

**FIGURE 8:**
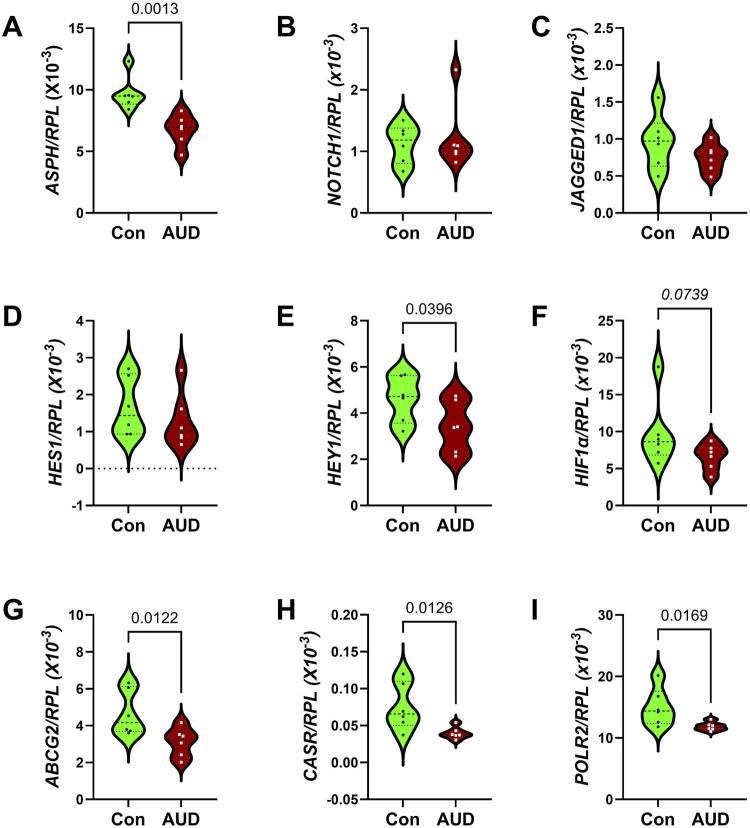
AUD impacts Notch pathway gene expression. Notch-related pathway mRNA transcripts were measured frontal lobe tissue from 6 AUD and 6 control donors using a custom multiplex magnetic bead-based RNA hybridization assay (see Methods). Violin plots (mean, quartiles, individual points) display inter-group differences in the levels of gene expression corresponding to **(A)** *ASPH*, **(B)** *NOTCH1*, **(C)** *JAGGED1*, **(D)** *HES1*, **(E)** *HEY1*, **(F)** *HIF1α*, **(G)** *ABCG2*, **(H)** *CASR*, and **(I)** *POLR2*, with results normalized to *RPL13a*. Data were analyzed by two-way ANOVA (Table 2) and *post hoc* multiple comparisons tests. Significant (p ≤ 0.05) and statistical trend-wise differences (italicized; 0.10 < p < 0.05) are shown within the panels. Also see Figure 4C-Heatmap.

Notch pathway genes were evaluated because preclinical studies linked chronic heavy ethanol consumption and inhibition of ASPH and Notch pathway mRNA expression to WM atrophy and degeneration [35]. Multiplex mRNA hybridization assays measured *ASPH, NOTCH1, JAGGED1, HES1, HEY1, HIF1α, ABCCG2, CASR* and *POLR2a,* with results normalized to RPL13. Two-way ANOVA detected significant effects of AUD and Biomarkers on the expression of Notch pathway genes, but no AUD x biomarker interactive effects (Table 2). Post hoc multiple comparison tests detected significantly reduced levels of *ASPH* (Figure 8A), *HEY1* (Figure 8E), *ABCG2* (Figure 8G), *CASR* (Figure 8H), and *POLR2* (Figure 8I), and a trendwise reduction in HIF1α (Figure 8F) in AUD samples. In contrast, *NOTCH1* (Figure 8B), *JAGGED1* (Figure 8C)*, and HES1* (Figure 8D) genes were similarly expressed in AUD and control samples. The corresponding heatmap, which includes the HPRT1 control gene, illustrates the intra- and intergroup differences in gene expression, along with the significant or trendwise effects of AUD (Figure 4C). Notably, except for *HPRT1*, genes with the lowest abundance showed no intergroup differences, suggesting that factors responsible for higher levels of gene expression were most significantly impacted by chronic alcohol exposure.

## Discussion

White matter degeneration, a consistent feature of ARBD, contributes to cognitive-motor impairment, yet its pathogenesis is poorly understood. Evidence suggests that WM atrophy in AUD is mediated by myelin and fiber loss due to ethanol’s targeting of oligodendrocytes. To better understand the mechanisms by which chronic alcohol misuse targets cells responsible for producing and maintaining CNS myelin and leads to WM degeneration, we characterized oligodendrocyte-related molecular and biochemical pathologies in postmortem AUD brains. The research focused on the BA8/9 anterior frontal brain region because it is a target of ARBD. Correspondingly, histopathological studies demonstrated variable degrees of myelin loss in anterior frontal lobe WM, consistent with previous reports [6]. The biochemical histopathologic correlates revealed by imaging mass spectrometry, consist of altered expression of myelin sphingolipids and phospholipids, but significantly greater reductions in sphingolipid populations as summarized in Supplementary Figure S1, from an earlier study [18]. Sphingolipids have a role in cognition and function. Functional impairments due to WM degeneration in BA8/9 account for deficits related to the frontal eye fields, short-term, spatial, semantic, perceptual, and emotional recognition memory, verbal fluency, attention, and executive functions [49] in AUD [50].

To investigate AUD’s effects on oligodendrocyte function, oligodendrocyte/myelin glycoprotein immunoreactivity and mRNA expression were measured by duplex ELISAs and multiplex RNA hybridization assays. The results demonstrated significant differences in oligodendrocyte/myelin glycoprotein expression, mainly in the form of increased levels in samples from the AUD cases. Both approaches yielded similar results in that AUD was associated with significantly increased expression of both immature and mature oligodendrocyte/myelin glycoproteins, as well as GFAP, a marker of astrocytes. In a previous study of human AUD, increased MAG and GFAP were also detected in the brain [51]. In contrast, molecules such as Nestin, vimentin, and the KLK6 and KLK8 serine proteases, which have broad functions during development, myelination, and synaptic plasticity [52], were unaffected by AUD. The higher levels of oligodendrocyte glycoprotein expression in AUD suggest compensatory responses related to myelin loss and possibly dysregulated oligodendrocyte function. To some degree, the abnormalities detected in human AUD brains mimic the findings in experimental models. The main difference is that in the human cases, the upregulated expression of oligodendrocyte/myelin glycoproteins was broad, whereas in the experimental models, the immature oligodendrocyte/myelin glycoproteins were mainly increased whereas the mature molecules were reduced by chronic ethanol exposure [53]. The primary conclusion is that AUD and experimental chronic ethanol feeding, accompanied by ARBD, significantly alter WM oligodendrocyte function, as evidenced by broad shifts in oligodendrocyte/myelin glycoprotein expression. Differential responses in human brains versus experimental animal models with ARBD could be linked to various human lifestyle co-factors such as smoking, nutrition, and variability in the rates, duration, consistency, and duration of alcohol misuse.

Although neuroinflammation is generally regarded as a significant driver of ARBD, the broad survey of proinflammatory cytokines and chemokines, and anti-inflammatory cytokines yielded largely negative results. The one exception was the finding of significantly reduced IL-10 in AUD samples. Since IL-10 is neuroprotective against neurodegeneration, its reduced levels may have contributed to chronic WM atrophy and degeneration of oligodendrocytes. This finding is consistent with a previous report showing reduced anti-inflammatory effects of chronic ethanol exposure [54]. Other studies linking increased neuroinflammatory responses to alcohol were largely related to short-term, binge, or developmental exposures [54–57] whereas chronic ethanol consumption was associated with immune or cytokine suppression [58, 59]. In essence, the potential role of persistent chronic neuroinflammation as a mediator of WM ARBD was not demonstrated in this study.

Trophic factors are important mediators of neuronal, glial, and vascular growth and function. AUD-associated significant or trendwise reductions in HGF and VEGF could reflect critical adverse effects of chronic ethanol exposure on CNS vascular function, which was not specifically investigated. These results contrast with data showing that low or moderate alcohol consumption provides cerebrovascular protection due to pro-angiogenic and anti-inflammatory effects [60, 61]. Nonetheless, together with the upregulated expression of GFAP reflecting astrocytic dysfunction that has been associated with loss of blood-brain barrier (BBB) integrity [62, 63], reduced expression of VEGF and HGF may have relevance to the disruption that occurs in ARBD [60].

AD biomarkers were examined due to a strong interest in the potential contributions of AD as a mediator of ARBD [64]. No significant or trendwise effects of AUD were observed with respect to any of the standard AD biomarkers, corresponding with previous studies in experimental models [59], but discordant with other studies in which adverse effects of alcohol were detected in genetic rather than sporadic models of AD [65]. Regarding the present study, it is noteworthy that the mean ages of the AUD and control subjects were in the mid-50s, ranging from 40 to 67, which is relatively young for AD. Whether AD-related abnormalities would emerge later with prolonged survival of people with AUD is unknown. The main conclusion from this study is that AD pathology does not appear to have contributed to WM neurodegeneration at these middle-age stages of AUD/ARBD.

Impairments in CNS insulin and IGF signaling networks have been well documented in experimental models of ARBD [22, 23]. In contrast, comparable studies in humans with AUD are scant. In this report, the findings of reduced mRNA levels of insulin and IGF trophic factors and receptors are consistent with an earlier publication utilizing different methodology to measure gene expression in human postmortem brain tissue with ARBD [51]. In addition, using more sensitive methods, we detected a statistical trendwise reduction in IRS1 mRNA in AUD, which was not previously observed. Together, these independent analyses support the concept that ARBD in humans is associated with impairments in CNS insulin and IGF signaling, as observed in experimental models of chronic ethanol feeding [66, 67]. However, multiplex ELISAs to examine AUD effects on related downstream signaling molecule protein and phosphoprotein expression yielded broadly negative results, which contrast with previous findings in experimental models [9, 59]. A likely explanation for the discordant results was the poor preservation of protein phosphorylation in postmortem human tissue.

Notch pathway gene expression was measured because previous studies showed that: 1) chronic ethanol exposure inhibits Notch signaling and ASPH expression via impairments in insulin/IGF signaling in the brain [68]; 2) ASPH regulates Notch [32, 34]; 3) ASPH is expressed in WM [36] and its levels are reduced by alcohol exposure [35]; and 4) Notch has critical roles in WM oligodendrocyte functions including myelin synthesis and maintenance [30, 69]. Beyond research on cerebral autosomal dominant arteriopathy with subcortical infarcts and leukoencephalopathy (CADASIL), which is caused by Notch3 mutations [36, 61], little is known about the effects of downregulated or impaired Notch signaling in adult brains on neurodegeneration. However, as in CADASIL, ARBD WM pathology is characterized by myelin loss and oligodendrocyte dysfunction. The present study links AUD’s inhibitory effects on WM/oligodendrocyte integrity with inhibition of ASPH and Notch pathway signaling. The aggregate findings suggest that AUD with WM ARBD is mediated by impaired insulin/IGF signaling, which leads to the inhibition of ASPH. Attendant impairment of Notch networks compromises oligodendrocyte functions needed to synthesize and maintain CNS WM myelin, thereby contributing to cognitive and motor dysfunctions associated with AUD/ARBD.

In a recent related study of the same cases evaluated herein, we investigated the effects of AUD on the molecular and biochemical pathology in the cerebellar vermis [37], which, like anterior frontal WM, is a major target of ARBD. The results of these parallel studies demonstrated similarly broad AUD effects on oligodendrocyte glycoprotein immunoreactivity and mRNA expression and minimal modulation of cytokines or chemokines in both the frontal lobe and cerebellum. GFAP was increased in the frontal lobe and not in the cerebellum. HGF was inhibited by AUD in both regions; however, in the frontal lobe only, VEGF and SDF were inhibited, whereas SCF was modestly increased by AUD. AUD inhibited insulin/IGF/IRS mRNAs in the frontal lobe but had no significant or trendwise effects in the cerebellum. On the other hand, AUD was associated with increased relative tyrosine phosphorylation of the IGF-1R in both structures, but inhibition of Akt and GSK-3β only in the cerebellum. Although AUD inhibited ASPH expression in both the frontal lobe and cerebellum, the downstream consequence linked to ASPH’s regulatory role in Notch signaling was to inhibit HEY1 transcription factor in the frontal lobe and HES1 in the cerebellum. Furthermore, several other Notch network-related genes were inhibited by AUD in the frontal lobe, but not in the cerebellum. Therefore, although AUD-related WM pathology in both the frontal lobe and cerebellum is linked to dysregulation of oligodendrocyte function, the mechanisms underlying these dysfunctions are not identical. In the frontal lobe, AUD-related WM degeneration was likely mediated by GFAP activation, and inhibition of ASPH-HEY1 and Notch network molecules, insulin/IGF/IRS pathway genes, and trophic factors including HGF, VEGF, and SDF, whereas in the cerebellum, the main effects of AUD were to inhibit Akt/GSK signaling, ASPH-HES1 expression, and HGF. Together, these findings suggest that strategies to reduce or reverse AUD-related white matter degeneration will likely require multi-targeted approaches.

## Data Availability

The datasets created and/or analyzed in this study are available from the corresponding author upon reasonable request.

## Footnotes

1
www.qiagen.com/handbooks

2
https://www.rndsystems.com/resources/protocols/how-run-rd-systems-luminex-assay?_gl=1*3qufyf*_up*MQ.*_gs*MQ.&gclid=Cj0KCQiArt_JBhCTARIsADQZaylimkKqpfj4YoGhU01ocFN_AFyNAJWU9eHU5uzdOeAhP7ByxbDpdkIaAtcWEALw_wcB&gclsrc=aw.ds&gbraid=0AAAAAD_kmX3Qz6TNbEqV0_4hqgJ3sD-eP

3
https://documents.thermofisher.com/TFS-Assets/LSG/brochures/defining-quantigene-plex-assay-panel-app-note.pdf
