# Supplementary material for: Molecular and biochemical correlates of frontal lobe white matter degeneration in humans with alcohol use disorder
Source: Adv Drug Alcohol Res. 2026 Feb 24;6:15431. doi: 10.3389/adar.2026.15431 (PMC12971536; doi:10.3389/adar.2026.15431)
Supplement: Supplementary file 1 [file Supplementaryfile1.docx]

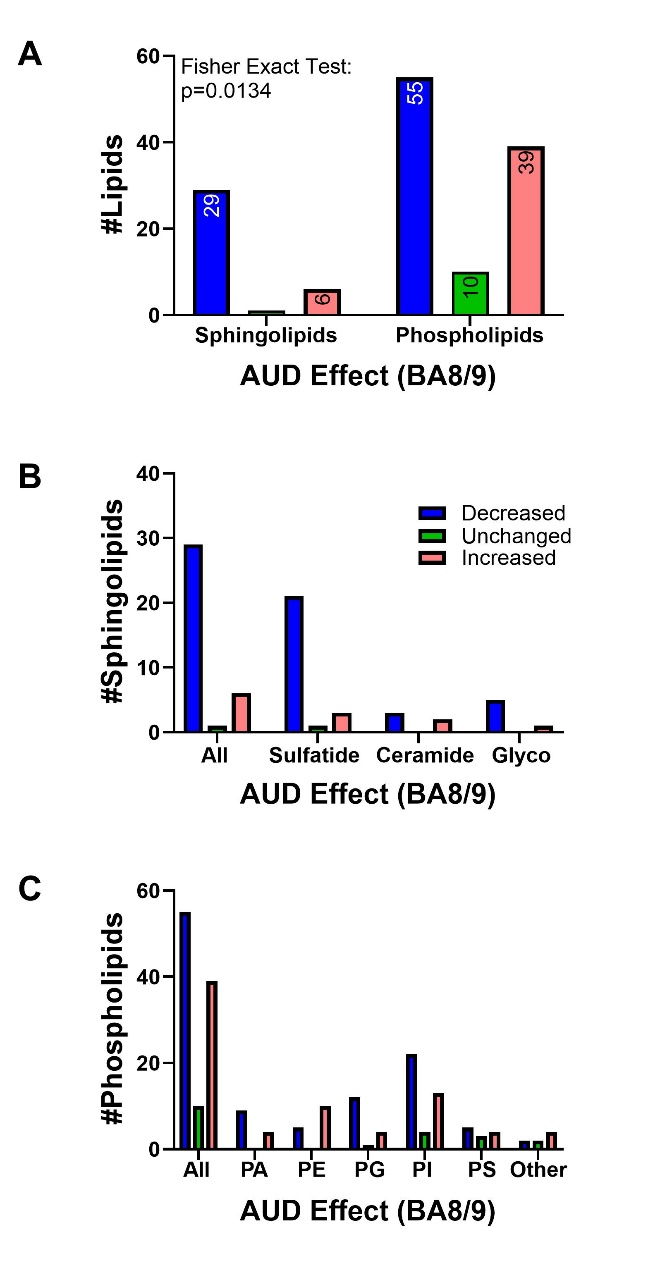


Supplementary Figure 1: Matrix-assisted desorption ionization imaging mass spectrometry analysis was used to measure and characterize sphingolipid and phospholipid profiles in fresh frozen postmortem anterior frontal lobe white matter of human control and alcohol use disorder (AUD) participants. (A) The summary results, adopted from an earlier publication (de la Monte, et al 2018), demonstrate disproportionate reductions in sphingolipid compared with phospholipid expression in AUD versus control white matter. The numbers in the bars correspond to the number of each lipid subtype detected in the samples. (B) The most notable AUD-associated reductions in sphingolipids pertained to sulfatide rather than ceramide or glycosphingolipids. (C) AUD-associated phospholipid changes were relatively balanced in terms of reductions versus increases in expression.

*de la Monte SM, Kay J, Yalcin EB, Kril JJ, Sheedy D, Sutherland GT: Imaging mass spectrometry of frontal white matter lipid changes in human alcoholics. Alcohol 2018, 67:51-63*


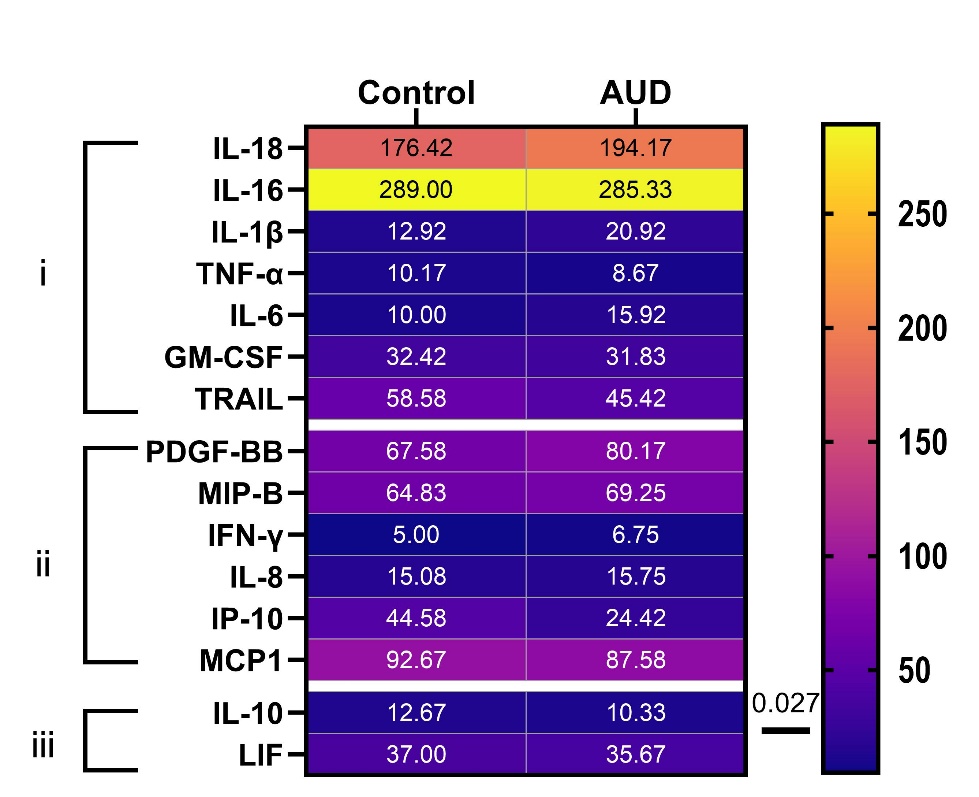


Supplementary Figure 2: Heatmap display of (i) proinflammatory cytokine, (ii) proinflammatory chemokine, and (iii) anti-inflammatory cytokine expression in human control (n=6) and alcohol use disorder (AUD; n=6) anterior frontal lobe white matter. The cytokine/chemokine molecules were measured in tissue homogenates using commercial magnetic bead-based multiplex ELISAs (see Stable 4 for cytokine/chemokine abbreviation definitions). Immunoreactivity was normalized to protein content. The results displayed correspond to fluorescent light units of cytokine immunoreactivity per 100µg brain tissue protein. The data were analyzed by two-way ANOVA (see Table 2) with post hoc multiple comparisons. The only significant difference was lower levels of IL-10 in AUD compared to the control (**p < 0.01).


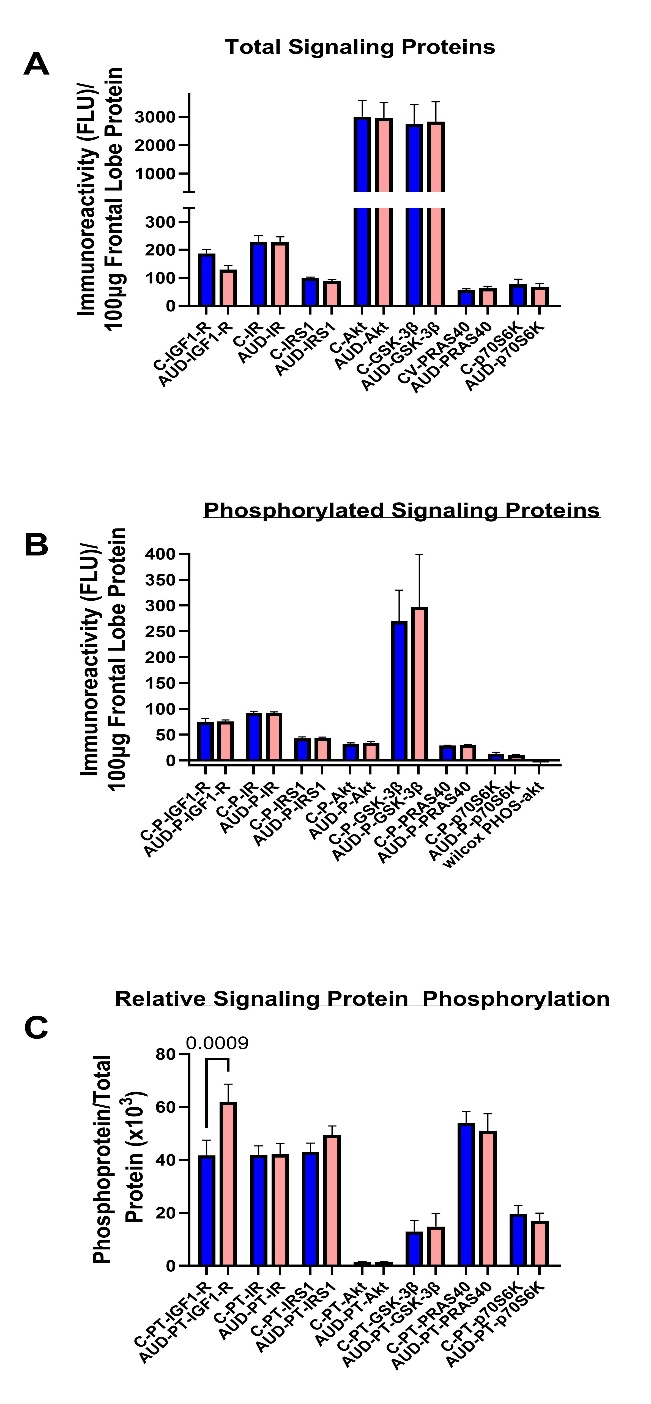
Supplementary Figure 3: Human postmortem anterior frontal lobe white matter tissue homogenates from control (blue bars) and AUD (orange bars) participants were analyzed with commercial 7-plex (A) Akt and (B) phospho-Akt magnetic bead-based ELISA panels, and the results were used to calculate (C) relative levels of protein phosphorylation (PT) (see Stable 5 for abbreviation definitions). Immunoreactivity was normalized to protein content. The results displayed correspond to fluorescent light units (FLU)/100µg frontal lobe protein. Graphs depict mean ± S.D. of results. The data were analyzed using a two-way ANOVA (see Table 2) with post hoc multiple comparisons. The only significant difference was the higher mean level of ^pYpY1135/1136^IGF1-R/total IGF1-R in ALC-FL compared to the control.
