# Supplementary material for: Molecular and biochemical correlates of frontal lobe white matter degeneration in humans with alcohol use disorder
Source: Adv Drug Alcohol Res. 2026 Feb 24;6:15431. doi: 10.3389/adar.2026.15431 (PMC12971536; doi:10.3389/adar.2026.15431)
Supplement: Supplementary file 2 [file Table1.docx]

**Supplementary Table 1: Reagents and Instrument Sources**

| **Reagents** | **Commercial Source** |
| --- | --- |
| Bicinchoninic acid reagents, secondary HRP-conjugated antibodies, Superblock-TBS, MaxiSorp 96-well plates | ThermoFisher Scientific, Bedford MA USA |
| Amplex UltraRed soluble fluorophore, 4- Methylumbelliferyl phosphate (4-MUP) | Invitrogen, ThermoFisher, Bedford MA, USA |
| Alkaline phosphatase streptavidin, Proton Biotin Protein Labeling Kit | Vector Laboratories Inc, Newark CA USA |
| Miscellaneous fine chemical reagents | CalBiochem/Millipore Sigma, Burlington, MA, USA; Pierce Chemical, Dallas, TX, USA; or Sigma-Aldrich Co., St. Louis, MO, USA |
| **Multiplex Panels** |  |
| Total and phospho-Akt/mTOR Multiplex panels | Millipore-Sigma, Bedford, MA USA |
| 5-Plex MILLIPLEX MAP Human Cytokine Magnetic Bead Panel | Millipore-Sigma, Bedford, MA USA |
| 11-Plex and 10Plex MILLIPLEX MAP Human Cytokine Magnetic Panels | Millipore, Burlington, MA, USA |
| Total 7-Plex and phospho Akt Magnetic 7-Plex Panel; LHO0001M | ThermoFisher/Invitrogen, Camarillo, CA, USA |
| QuantiGene 2.0 Plex Set Cat# 312185, Custom Human Glial 10-Plex Panel | Affymetrix Inc., Santa Clara, CA, USA |
| QuantiGene 2.0 Plex Set Cat# 312177, Custom Human Insulin/Notch Pathway-20-Plex Panel | Affymetrix Inc., Santa Clara, CA, USA |
|  |  |
| **Instruments** | **Commercial Source** |
| Luminex MAGPIX | Diasorin, Austin TX, USA |
| SpectraMax M5 microplate reader | Molecular Devices, San Jose, CA USA |
| TissueLyser II | The Cavey Laboratory Engineering Co., Guildford, Surrey, UK |
| Eppendorf epMotion 330 | Framingham, MA, USA |
| Spectra-Max M5 Multimode Plate Reader | Molecular Devices, Sunnyvale, CA, USA |
