# Supplementary material for: Molecular and biochemical correlates of frontal lobe white matter degeneration in humans with alcohol use disorder
Source: Adv Drug Alcohol Res. 2026 Feb 24;6:15431. doi: 10.3389/adar.2026.15431 (PMC12971536; doi:10.3389/adar.2026.15431)
Supplement: Supplementary file 4 [file Table2.docx]

**Supplementary Table 2: Commercial Antibodies Used in Duplex ELISAs Including RRID #s**

| **Antibody Target abbreviation** | **Antibody Target (full name)** | **Source** | **Monoclonal/Polyclonal** | **Stock (mg/mL)** | **Final Dilution (µg/mL)** | **Commercial Source** | **Reference*** | **RRID** |
| --- | --- | --- | --- | --- | --- | --- | --- | --- |
| AβPP | Amyloid β-Precursor Protein | Rabbit | Polyclonal | 0.197 | 0.246 | Cell Signaling, Danvers, MA | Cat. #2452 | **AB_10694227** |
| Aβ_1-42_ | Amyloid β Peptide, (1-42) | Mouse | Monoclonal | 0.2 | 0.4 | Santa Cruz Biotechnology, Dallas, TX, USA | Cat. #sc-28365 | **AB_626669** |
| Tau | Tubulin-associated unit | Rabbit | Polyclonal | 6.2 | 3.1 | Agilent/Dako, Santa Clara, CA | REF A0024 | **AB_10013724** |
| pTau (PHF13; Ser396) | Phosphorylated Tau | Mouse | Monoclonal | 1.21 | 0.40 | Cell Signaling, Danvers MA | #9632 | **AB_2266237** |
| HNE | 4-hydroxynonenal | Goat | Polyclonal | 0.8 | 1.6 | Abcam, Boston, MA | ab46544 | [**AB_722493**](https://www.google.com/search?q=RRID+is+AB_722493&mstk=AUtExfCdHpCeH7jxIyDNjCUKFvQPlz3TVP7zEsXtq2w9SJhE7YZe814ANlxrrYKK4uHZVtM1qJPD0KEd86TXf6beWE-LpPHY-H8GQjGMYW8Fy9zWVqMXK0ndAcnPpv07wCMETM65qmAPTVYQkA0esI0GefO5Is9YCHYBBUvWOYeeDKXR_-M&csui=3&ved=2ahUKEwiZp4ehuLGRAxV138kDHZovG2kQgK4QegQIARAE) |
| Ubiquitin | Ubiquitin | Rabbit | Polyclonal | 0.25 | 0.5 | Abcam, Boston, MA | ab7780-500 | **AB_2313773** |
| CNPase (11-5B) | 2',3'-cyclic nucleotide 3' phosphodiesterase | Mouse | Monoclonal | 1.0 | 2.0 | Abcam, Boston, MA | ab6319 | AB_2082593 |
| GALC protein; GC-globulin) | Group-specific component Vitamin D Binding | Rabbit | Polyclonal | 1.0 | 2.0 | Abcam, Boston, MA | ab83752 | AB_2108528 |
| MAG1 | Myelin-Associated Glycoprotein 1 | Mouse | Monoclonal | 0.5 | 0.25 | Abcam, Boston, MA | ab89780 | AB_2042411 |
| MOG | Myelin Oligodendrocyte Glycoprotein | Rabbit | Polyclonal | 1.0 | 2.0 | Abcam, Boston, MA | ab32760 | AB_2145529 |
| MBP | Myelin basic protein | Rabbit | Polyclonal | 1.0 | 2.0 | MilliporeSigma Burlington, MA | M3821 | AB_1841021 |
| PLP | Proteolipid Protein 1 | Rabbit | Polyclonal | Serum | 1:2000 | Abcam, Boston, MA | ab28486 | AB_776593 |
| PDGFRA | Platelet-derived growth factor receptor, alpha polypeptide | Rabbit | Polyclonal | 1.0 | 1.0 | Abcam, Boston, MA | ab61219 | AB_2162341 |
| Nestin | Nestin | Rabbit | Polyclonal | Serum | 1:2000 | Abcam, Boston, MA | ab27952 | AB_776698 |
| Vimentin | Vimentin | Mouse | Monoclonal | 1.0 | 2.5 | Abcam, Boston, MA | ab8978 | AB_306907 |
| GFAP | Glial Fibrillary Acidic Protein | Goat | Polyclonal | 0.5 | 0.5 | Abcam, Boston, MA | ab53554 | AB_880202 |
| RPLPO | Large acidic ribosomal protein | Mouse | Monoclonal | 1.0 | 0.1 | Proteintech, Chicago, IL | AG1829 | AB_2254064 |

*RRID= Research Resource Identifier
