# Supplementary material for: Molecular and biochemical correlates of frontal lobe white matter degeneration in humans with alcohol use disorder
Source: Adv Drug Alcohol Res. 2026 Feb 24;6:15431. doi: 10.3389/adar.2026.15431 (PMC12971536; doi:10.3389/adar.2026.15431)
Supplement: Supplementary file 5 [file Table5.docx]

| **Insulin/IGF-Akt Pathway Molecules** | **Protein** | **Phosphoprotein** |
| --- | --- | --- |
| Insulin Receptor | Insulin-R | ^pYpY1162/1163^-Insulin R |
| Insulin-Like Growth Factor Receptor Type 1 | IGF1-R | ^pYpY1135/1136^-IGF1-R |
| Insulin Receptor Substrate, Type 1 | IRS1 | ^pS636^-IRS1 |
| Akt (Protein Kinase B) | Akt | ^pS473^-Akt |
| Glycogen Synthase Kinase 3β | GSK-3β | ^pS9^-GSK3β |
| p70 Ribosomal S6 kinase | P70S6K | ^pT412^-p70S6K |
| Ribosomal Protein S6 | RPS6 | ^pS235/S236^-RPS6 |

**Supplementary Table 5: 7-Plex Akt Pathway Proteins and Phosphoproteins**
