# Supplementary material for: Molecular and biochemical correlates of frontal lobe white matter degeneration in humans with alcohol use disorder
Source: Adv Drug Alcohol Res. 2026 Feb 24;6:15431. doi: 10.3389/adar.2026.15431 (PMC12971536; doi:10.3389/adar.2026.15431)
Supplement: Supplementary file 6 [file Table3.docx]

**STable 3: Glial Molecules and Their Functions**

| Abbreviation | Full Name | Gene Names | Main Cellular Expression | Product Functions |
| --- | --- | --- | --- | --- |
|  |  |  |  |  |
| CNP | 2',3'-cyclic nucleotide 3' phosphodiesterase | CNPase, EC, *CNP1* | All oligodendrocytes | Myelin-associated marker of oligodendrocytes and Schwann cells that likely play important roles in myelin membrane development and maintenance of axonal integrity. |
| *CSPG4* | Chondroitin Sulfate Proteoglycan 4 | *MCSP;* Melanoma-Associated Chondroitin Sulfate | Oligoprogenitor cells/ immature oligodendrocytes | A major extracellular matrix molecule implicated in shaping O2A oligodendrocyte precursor cells; inhibits migration and differentiation of O2A and inhibits oligodendrocyte myelination |
| *KLK6* | Kallikrein-related peptidase 6; neurosin | *KLK6* | Mature myelinating oligodendrocytes | Trypsin-like Serine protease expressed in oligodendrocytes, implicated in demyelination; degrades extracellular matrix components involved in synaptic maturation and plasticity in the brain |
| *KLK8* | Kallikrein-related peptidase 8/ | *KLK8* | Mature myelinating oligodendrocytes | Expressed in limbic structures linked to learning and memory; activated through N-methyl-d-aspartate receptors and dual-specific MEK1. Involved in hippocampal memory; upregulated in oligodendrocytes in response to injury and associated with myelin degradation. |
| GALC | Galactosylceramidase; Group-specific component Vitamin D Binding protein; Gc-globulin | *GALC; Galactosyceramidase* | Immature and mature myelinating oligodendrocytes | Multifunctional member of the albumin family, found in plasma, ascites fluid, cerebrospinal fluid, and on cell surfaces. Binds Vitamin D and plasma metabolites and transports them to target tissues. Encodes a lysosomal protein that hydrolyzes galactose ester bonds of major myelin lipids. |
| PDGFRα | Platelet Derived Growth Factor Receptor, alpha polypeptide | *PDGFRA;* CD140a | Oligoprogenitor cells/ immature oligodendrocytes | Cell surface tyrosine protein kinase receptor needed for embryonic skeletal development. Functions as an oligodendrocyte progenitor cell survival factor in white matter. |
| PLP1 | Proteolipid Protein 1 | *PLP1*, SPG2 | Premyelinating and immature myelinating oligodendrocytes | A major CNS myelin transmembrane protein involved in compaction, stabilization, and maintenance of myelin sheaths, oligodendrocyte development, and axonal survival. |
|  |  |  |  |  |
| MAG | Myelin Associated Glycoprotein | *MAG* | Myelinating and mature oligodendrocytes | Glycoprotein expressed on oligodendrocytes and Schwann cells that facilitates sialic acid- dependent cell-cell interactions between neuronal and myelinating cells. |
| MOG | Myelin Oligodendrocyte Glycoprotein | *MOG* | Myelinating and mature oligodendrocytes | Expressed on the surface of oligodendrocytes and the outer surface of myelin sheaths. MOG may help mediate the completion or maintenance of myelin sheaths. |
| MBP | Myelin Basic Protein | *MBP* | Myelinating and mature oligodendrocytes | This major component of myelin sheaths in oligodendrocytes and Schwann cells aids in the formation and stabilization of myelin membranes. |
|  |  |  |  |  |
| NES | Nestin | *NES* | Reactive astrocytes following injury; neuroprogenitor cells | Intermediate protein that promotes the disassembly of phosphorylated vimentin during mitosis, and is required for mitogen-stimulated proliferation, survival, and renewal of neural progenitor cells. Up-regulated in reactive astrocytes following CNS injury |
| VIM | Vimentin | *VIM* | Specific marker for astrocytes and glial precursor cells before GFAP expression | Class-III intermediate filament that maintains cell shape and cytoplasm integrity, stabilizing cytoskeletal interactions. May have functional roles in peripheral nerve myelination. |
| GFAP | Glial Fibrillary Acidic Protein | *GFAP* | Astrocytes, mature | Major, Class III intermediate filament; cell-specific marker; cytoskeletal protein expressed in astrocytes. |
|  |  |  |  |  |
| *HPRT1* | Hypoxanthine phosphoribosyltrans-ferase 1 | *HPRT* | All cell types | Housekeeping gene regulates purinergic signaling in pluripotent stem cells |
| HSP70 | Heat Shock Protein 70 | *HSPA1A* | All cell types | Family of conserved, ubiquitously expressed HSPs; has a crucial cytoprotective role and is upregulated by a broad range of stresses. |
| *RPL13A* | Ribosomal Protein L13a | *RPL13A* | All cell types | Encodes a member of the L13P family of ribosomal proteins that is a component of the 60S subunit |
| RPLPO | Large acidic ribosomal protein | *RPLP1* | All cell types | Housekeeping gene encodes a component of 60S subunit and used for normalizing RT-qPCR results |
|  |  |  |  |  |

https://www.biocompare.com/Editorial-Articles/590587-A-Guide-to-Oligodendrocyte-Markers/
