# Supplementary material for: Molecular and biochemical correlates of frontal lobe white matter degeneration in humans with alcohol use disorder
Source: Adv Drug Alcohol Res. 2026 Feb 24;6:15431. doi: 10.3389/adar.2026.15431 (PMC12971536; doi:10.3389/adar.2026.15431)
Supplement: Supplementary file 7 [file Table4.docx]

**Supplementary Table 4: Cytokines and Trophic Factors-Multiplex ELISAs**

| **Factor** | **Full Name** |
| --- | --- |
| **Pro-inflammatory Cytokines** | |
| **IL-18** | Interleukin-18 |
| **IL-16** | Interleukin-16 |
| **IL-1β** | Interleukin-1beta; leukocyte pyrogen; leukocyte activating factor |
| **TNF-α** | Tumor necrosis factor-alpha; cachexin |
| **IL-6** | Interleukin-6 |
| **GM-CSF** | Granulocyte- Macrophage Colony stimulating factor; CSF-2 |
| **TRAIL** | Tumor necrosis factor‐related apoptosis‐inducing ligand |
| **Pro-Inflammatory Chemokines** | |
| **PDGF-BB** | Platelet-derived Growth Factor-BB |
| **MIP-1B** | Macrophage inflammatory protein 1 beta; Chemokine motif ligand 4 (CCL4) |
| **IFN-γ** | Interferon-gamma; type II interferon |
| **IL-8** | Interleukin-8 |
| **IP-10** | Interferon gamma induced protein 10; CXCL10 |
| **MCP-1** | Monocyte chemoattractant protein 1; CCL2 (chemokine motif ligand 2) |
| **Anti-Inflammatory Cytokines** | |
| **IL-10** | Interleukin-10; cytokine synthesis inhibitory factor |
| **LIF** | Leukemia inhibitory factor |
| **Trophic Factors** | |
| **b-FGF** | Basic fibroblast growth factor; FGF2 |
| **HGF** | Hepatocyte growth factor |
| **β NGF** | Beta-Nerve growth factor |
| **SCF** | Stem Cell Factor |
| **SDF 1α** | Stromal-derived factor 1alpha |
| **VEGF** | Vascular endothelial growth factor; vascular permeability factor (VPF) |
